# Cerebro-afferent vessel and pupillary basal diameter variation induced by stomatognathic trigeminal proprioception: a case report

**DOI:** 10.1186/1752-1947-6-275

**Published:** 2012-09-03

**Authors:** Vincenzo De Cicco

**Affiliations:** 1 Department of Oral Science, University “G. d’Annunzio”, via dei Vestini 31, Chieti, 66100, Italy

## Abstract

**Introduction:**

A patient affected by asymmetric hemodynamics of cerebro-afferent vessels underwent duplex color scanner investigations in occlusal proprioceptive un- and rebalance conditions. Pupillometric video-oculographic examinations were performed in order to spot connected trigeminal proprioceptive motor patterns able to interfere on sympathetic autonomic activity. The aim of this case report is to verify if involuntary jaw closing during swallowing, executed in unbalance and rebalance myoelectric activity, would be able to modify cerebral hemodynamics.

**Case presentation:**

A 56-year-old Caucasian Italian woman affected by asymmetric blood flow of cerebro-afferent vessels underwent an electromyographic investigation of her occlusal muscles in order to assess their occlusal functional balance. The extreme asymmetry of myoelectric activity in dental occlusion evidenced by electromyographic values suggested the rebalancing of the functions of occlusal muscles through concurrent transcutaneous stimulation of the trigeminal nerve supra- and submandibular motor branches. The above-mentioned method allowed the detection of a symmetric craniomandibular muscular relation that can be kept constant through the use of a cusp bite modeled on the inferior dental arch: called orthotic-syntropic bite for its peculiar use of electrostimulation. A few days later, the patient underwent a duplex color scanner investigation and pupillometric video-oculographic examinations in occlusal unbalance and rebalance conditions.

**Conclusions:**

A comparative data analysis showed that an unbalanced dental occlusal function may represent an interferential pattern on cerebral hemodynamics velocity and pupillometric evaluations have proved useful both in the analysis of locus coeruleus functional modalities and as a diagnostic tool in the assessment of pathologies involving locus coeruleus and autonomic systems. The inclusion of myoelectric masseter examinations can be useful in patients with asymmetric hemodynamics of cerebro-afferent vessels and dental occlusal proprioceptive rebalance can integrate the complex therapy of patients with increased chronic sympathetic activity.

## Introduction

Recent brain mapping studies have revealed the effects of gum chewing and voluntary dental tapping and clenching on brain function. An increasing flow of evidence has indicated that the neural activity of daily chewing consistently stimulates cerebral areas with positive effects in maintaining brain functions, associated with an increase of blood flow, supporting earlier evidence that not only the areas of cerebral cortex related to movements but also the hippocampus and prefrontal cortex, normally associated with memory, are activated by jaw movements [1-3]. Furthermore, an epidemiological survey and cross-sectional study with subjects whose ages ranged from 50 years to 80 years demonstrated that reduced chewing ability or dysfunctional teeth might induce senile processes with decrease of cognitive function and learning effect [4,5]. At the same time, findings on aged rats have demonstrated the effects generated by the loss of molar teeth with reduction of spatial memory, acetylcholine release from the parietal cortex [6] and alteration of the septohippocampal cholinergic system [7]. Yamazaki K. *et al*. verified that the number of extracted teeth was directly proportional to the loss of spatial memory and to the reduction of trkB-messenger ribonucleic acid (mRNA) levels [8].

Although the brain mapping study has high spatial resolution, it is unsuitable for a quantitative analysis of overall changes in cerebral blood flow associated with daily jaw chewing movements. Recent findings have utilized transcranial doppler ultrasonography to measure blood flow velocity in major cerebral blood vessels because this method provides the advantages of continuous real-time recording and ease-of-use allowing evaluation of relationships between jaw chewing and cerebral circulation [3,9]. The cited studies have correlated the change of task induced in cerebral blood flow due to the working side and to myoelectric intensity of the masseter muscle in voluntary activity. Given that chewing is a movement semi-automatically controlled by the brainstem generator pattern [10], the aim of this case report is to verify if involuntary jaw closing during swallowing, executed in unbalance and rebalance myoelectric activity, would be able to modify cerebral hemodynamics. Normally, dental occlusion takes place constantly at 1 minute intervals during swallowing and these occlusions are superior in number to voluntary chewing activity.

Pupillometry has proved useful to extend the autonomic relationships between trigeminal and vascular systems [11] because basal pupil diameter variations are correlated with the autonomic nervous system [12] and locus coeruleus (LC) activity [13]. The findings of Elam *et al*. [14], in fact, showed that sympathetic nerve activity is parallel to LC discharges. Also, the paragigantocellularis (PGi) nucleus of the ventral medulla is an important anatomic pathway which might mediate this relationship directly. The PGi is a critical relay for the sympathoexcitatory efferents of the autonomic hypothalamic centers that subserve both vascular muscular tone and reflexive pupillary dilation [14]. It presents afferent connections with LC and the trigeminal system [15]. Moreover, the pupillometric baseline closely tracks LC tonic discharge frequency and it is influenced by noradrenergic release ratio [13]. Neuroanatomical studies performed through an anterograde and retrograde transport method have indicated that many of the regions that received dense inputs from projected LC neurons, in turn, feed back to these coerulei neurons [16], which are uniformly sensitive to a variety of non-noxious stimuli, including tactile, visual, and auditory, with a specific degree of activation stimulus [17,18]. The trigeminal system is strictly connected with LC which exhibit mixed cellular elements with trigeminal mesencephalic neurons [16,19]. Couto *et al*. showed through anterograde and retrograde tract-tracing with fast blue injections reciprocal connections between the trigeminal and LC systems [20]. Moreover, Panneton *et al*., using anterograde transneuronal transport of the herpes simplex virus (HSV-1) into the anterior ethmoidal nerve, observed LC and PGi nuclei HSV-1-labeled [21]. At last, coerulean and peri-coerulean areas can be activated by increasing the discharge frequency of trigeminal mesencephalic neurons activated both by masseter spindle receptors due to excessive interocclusal space [22], and by periodontal receptors for increased occlusal charge [23]. In addition, the coerulean area can also be indirectly activated by the trigeminal motor nucleus. This nucleus does not have a definite nuclear delimitation but it is mixed with lateral reticular formation (LRF) parvocellular neurons [24], and it is part of the ascending reticular activating system [25]. Presumably, neuromotor hyperactivity of the mastication preferential side elicits a concomitant asymmetric brainstem stimulation of reward reticular systems [26], including diffused projection catecholaminergic systems of intermediate reticular formation nuclei (IRFn). Previous studies recorded short latency ipsilateral electrophysiological responses electrophysiological responses in LRF and IRFn after passive mandible dislocations [27]. Finally, the basal pupillometric test can be considered a non-invasive instrument of analysis apt to examine the responsivity of trigeminal proprioception in modeling both coerulean noradrenergic activity and autonomic sympathetic activity. The present case report documents basal pupillometric dynamics, with corneal topography, and blood flow velocity in carotid (C.a.) and vertebral (V.a.) arteries, performed through duplex color scanner examinations induced by involuntary jaw clenching during swallowing in unbalance and rebalance stomatognathic proprioception.

## Case presentation

**Figure 1:**
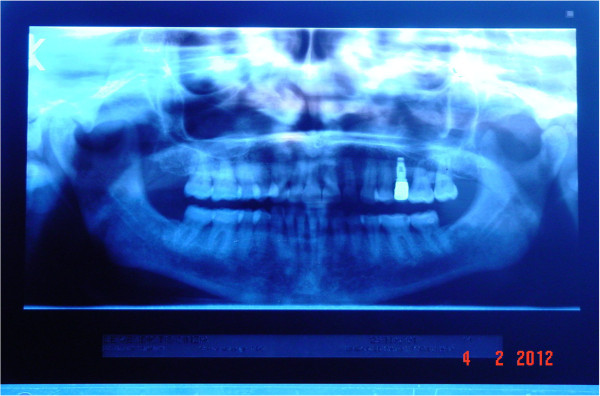
Orthopantomography: habitual occlusion with implant-dental prosthesis rehabilitation.

**Figure 2:**
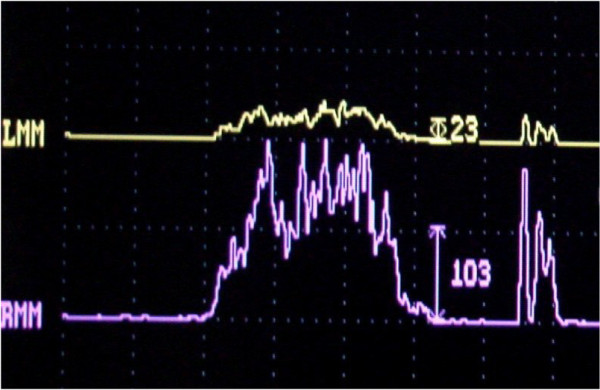
Electromyography values of masseters in habitual occlusion: the left myoelectric activity 23mV, the right myoelectric activity 103mV.

**Figure 3:**
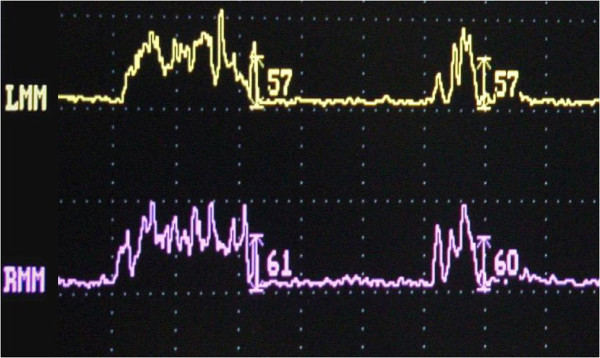
Electromyography values of masseters with orthotic-syntropic application: the left myoelectric activity 57mV, the right myoelectric activity 61mV.

The subject of our study, a 56-year-old Caucasian Italian woman, presents with an occlusal open bite and a complete dental formula, with only the left superior second premolar missing, substituted by an implantoprosthetic rehabilitation (Figure 1). The patient, a medical doctor, has a normal blood pressure range, is not affected by any metabolic disease and is a non-cigarette smoker. For the evaluation of her occlusal muscle activity, a bilateral electromyography (EMG) of her masseter muscle was recorded using an evaluation system of mandibular movement (K6-I; Myotronics, Seattle, WA, USA) and Duo-trode surface Ag-AgCl electrodes (Duo-trode; interelectrode distance: 19.5mm, Myotronics). EMG data were recorded at a sampling rate of 240Hz and amplified at a time constant of 0.06 seconds. For the evaluation of her muscle activity, voluntary dental clenching was executed and recorded during swallowing. In accordance with the dental diagnostic protocol [28], a preliminary evaluation of the patient’s myoelectric activity in dental occlusion was performed through muscle EMG in order to assess their functional balance. Registered values showed a remarkable functional asymmetry of masseter muscles, 23mV for her left masseter and 103mV for her right masseter (Figure 2). According to the expressed electromyographic values, muscular activity was symmetrized by applying a 15 minutes transcutaneous stimulation of trigeminal motor branches at low frequency for elevator occlusal muscles and at medium frequency for submandibular antagonist muscles. This method allowed detection of the functional trajectory of occlusal elevator muscles and to record a symmetric craniomandibular relation, positioning a self-hardening material between the dental arches. The same material was used to make a cusp bite modeled on the inferior dental arch named orthotic-syntropic bite for its peculiar use of electrostimulation. When the orthotic was applied, electromyographic control was repeated to verify occlusal myoelectric balance. Registrations have documented substantially equal values: 57mV for left masseter muscle and 61mV for right masseter (Figure 3). Immediately after, the patient was submitted to pupillometric and hemodynamic examinations in habitual occlusion first and with the orthotic soon after.

**Figure 4:**
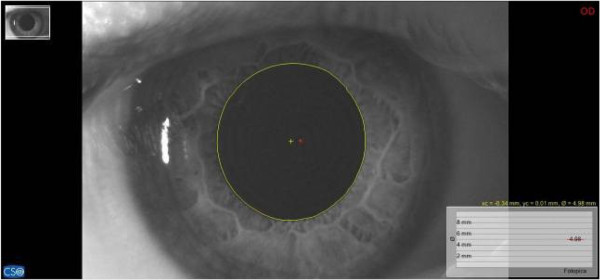
**Basal pupillometry in habitual occlusion.** Right pupil diameter: 4.98mm.

**Figure 5:**
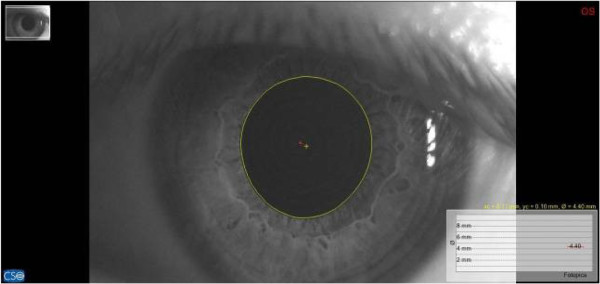
**Basal pupillometry in habitual occlusion.** Left pupil diameter: 4.40mm.

**Figure 6:**
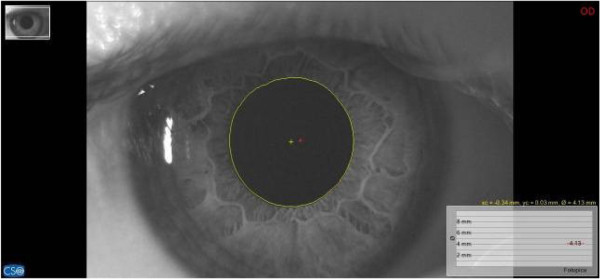
**Basal pupillometry with orthotic-syntropic application.** Right pupil diameter: 4.13mm.

**Figure 7:**
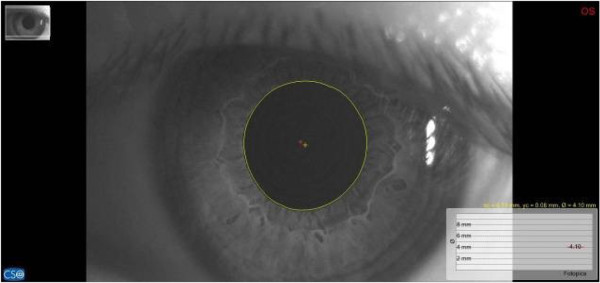
**Basal pupillometry with orthotic-syntropic application.** Left pupil diameter: 4.10mm.

For pupillary diameter measurement, we used a computerized corneal topographer MODI02 software 2005 LITE (CSO, Florence, Italy), made of a survey section by Placido disk 24 loops, camera sensor charge-coupled device (CCD) 1/3 inch and a claim support. The instrument presents, during the pupillar acquisition phases, a constant lighting of the disk and a 56mm distance of work. The points measured during data acquisition are 6.144, with a model elaboration higher than 100.000 points. Registered pupillometric analysis showed a remarkable right and left baseline asymmetry, respectively 4.98mm (Figure 4) and 4.40mm (Figure 5), whereas in the occlusal rebalance condition an equivalent pupil diameter was registered, 4.13mm right pupil (Figure 6) and 4.10mm left pupil (Figure 7). Indeed, pupillometric data analysis registered in occlusal rebalance shows a more suitable reduction of the basal diameter, with clear right side decrease, relating to higher occlusal myoelectric values.

**Figure 8:**
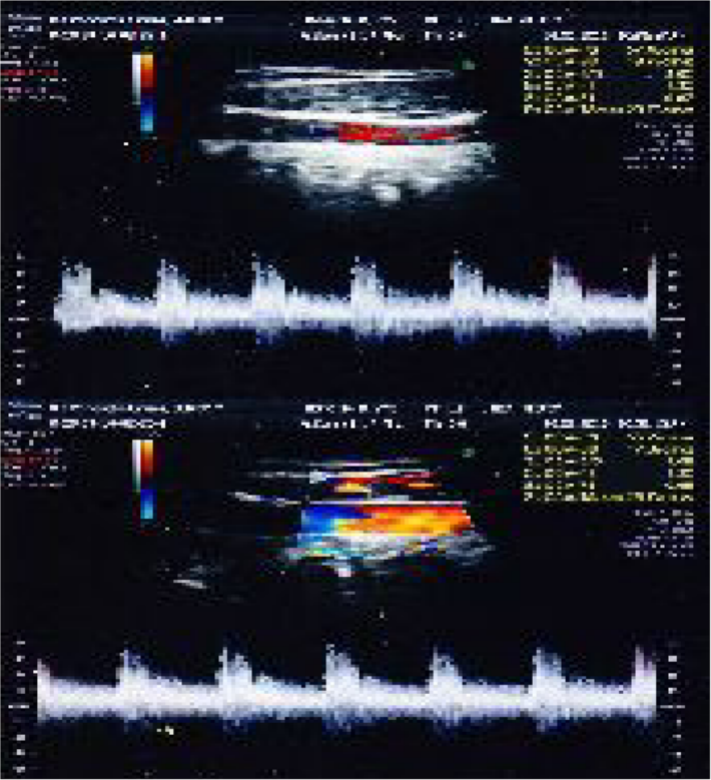
Left (up)/right (down) Carotid duplex color scanner recordings in habitual occlusion.

**Figure 9:**
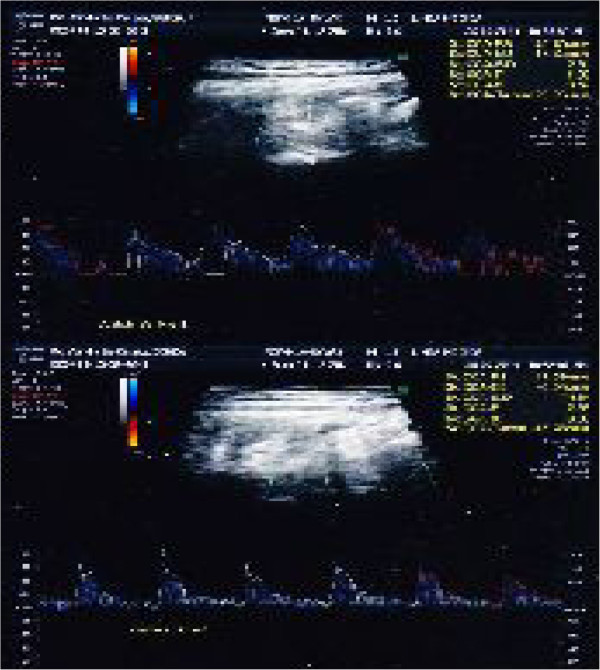
Left (up)/right (down) Vertebral duplex color scanner recordings in habitual occlusion.

**Figure 10:**
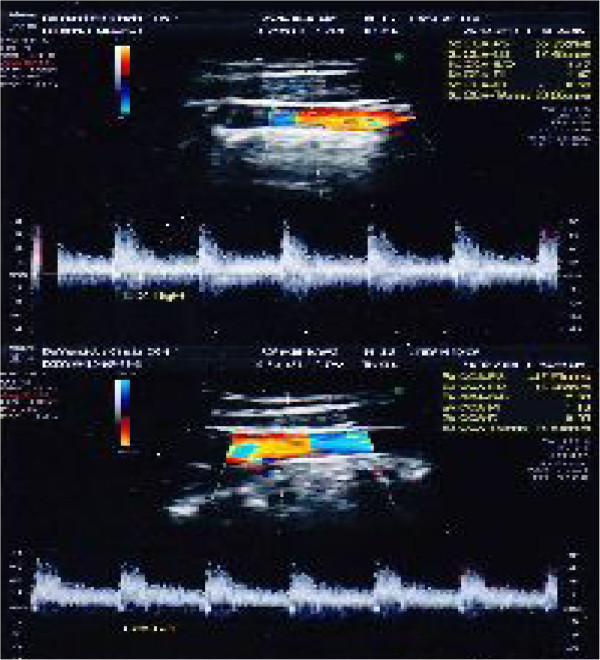
Left (up)/right (down) Carotid duplex color scanner recordings with orthotic-syntropic application.

**Figure 11:**
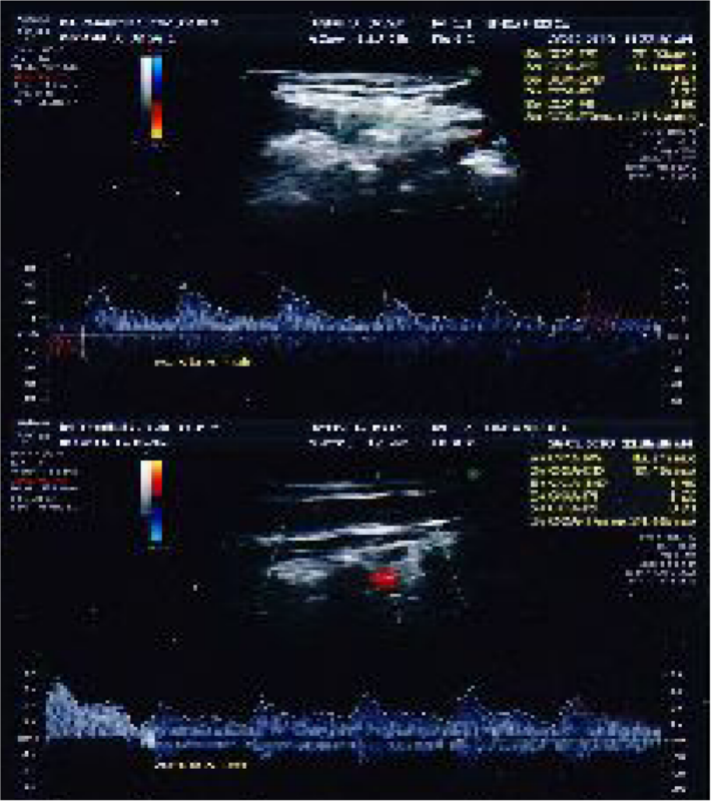
Left (up)/right (down) Vertebral duplex color scanner recordings with orthotic-syntropic application.

For blood flow computerized examination, a GE HealthCare echograph, Voluson E8 Expert model, was used, with a 3D-4D-color-power Doppler volumetric probe. The duplex color scanner investigations were executed with an interval of 60 minutes, in habitual occlusion first, (Figure 8 carotid artery, Figure 9 vertebral artery) and with the orthotic after (Figure 10 carotid artery, Figure 11 vertebral artery). The following evaluations were performed (see Table 1).

systolic pulsatility and average flow velocity: (**P.I.** Index);

systolic and diastolic relationship-flow: (**R.I.** Index);

systolic peak in cm/second: (**P.S.** Index);

diastasis cordis in cm/second: (**E.D.** Index);

systole-diastole relationship: (S-D Index);

Carotid artery: C.a.;

Vertebral artery: V.a.

**Table 1:** Hemodynamic variations of cerebro-afferent vessels

**Hemodynamic variations of cerebro-afferent vessels**								
	**OCCLUSAL UNBALANCE**				**ORTHOTIC APPLICATION**			
	**C.a.**	**C.a.**	**V.a.**	**V.a.**	**C.a.**	**C.a.**	**V.a.**	**V.a.**
	**Right**	**Left**	**Right**	**Left**	**Right**	**Left**	**Right**	**Left**
**PI** index	1.30	1.30	1.0	2.88	1.42	1.16	1.23	1.23
**RI** index	0.67	0.68	0.62	0.99	0.68	0.65	0.69	0.71
**PS** index	57.40	55.27	46.45	47.58	55.35	47.97	39.03	43.21
**ED** index	18.79	17.87	17.82	0.64	17.48	16.96	12.16	12.70
**S-D** index	3.05	3.09	2.61	74.34	3.17	2.83	3.21	3.40

systolic pulsatility and average flow velocity: (**P.I.** Index);

systolic and diastolic relationship-flow: (**R.I.** Index);

systolic peak in cm/second: (**P.S.** Index);

diastasis cordis in cm/second: (**E.D.** Index);

systole-diastole relationship: (S-D Index);

Carotid artery: C.a.;

Vertebral artery: V.a.

The registrations reveal that the patient’s left V.a. hemodynamic is more influenced by trigeminal proprioception. In fact, the orthotic application reduces on the left the S-D index of 70.94 and equilibrates the values of both vertebral arteries, 3.40 (left) and 3.21 (right), respectively. Whereas, in the ED index, diastolic flow increase of 12.06 cm/second of the left V.a. makes the values of both arteries equal, 12.70 (left) and 12.16 (right) respectively. Moreover, in the PI index it is possible to observe that the different average flow between the right (1.0) and left (2.88) vertebral arteries is totally cancelled in occlusal rebalance, with perfectly equal values (1.23). Also the PS Index confirms the previous results because a general reduction of hemodynamic values is registered both in carotid and vertebral arteries after orthotic application. In fact, the systolic hematic peak, expressed in cm/second, shows decreases of 2.05 on the right and of 7.69 on the left in the carotid arteries, while in vertebral arteries the decreases are of 7.42 on the right and of 4.37 on the left. The RI index does not seem to be influenced by occlusal proprioception.

## Discussion

There are reports in the literature of the effects on cerebral blood flow of voluntary chewing and of regional increases in brain neural activity, but the examinations described in the present case report show that the proprioceptive intensity during involuntary jaw occlusion in swallowing can also influence the hemodynamics in major cerebral blood vessels. The basal pupil diameter evaluations could be a valid method to connect the neurophysiologic relationships between proprioceptive trigeminal system and LC-sympathetic autonomic functional modes. The comparative analysis of the obtained results seem to confirm these relationships. In fact, increased right occlusal myoelectric expressivity (103mV) in our patient is in homolateral relation with her pupillary basal diameter (4.98mm) and both exceed the values registered contralaterally, 23mV and 4.40mm respectively. Contrary to expectation, it is her left vertebral artery that presents the most suitable hemodynamic variations and it is more influenced by sympathetic autonomic action. In coherence with what was registered in pupillometric analyses (4.13mm right pupil and 4.10mm left pupil), proprioceptive rebalance has determined, even in the cerebro-afferent vascular system, a general reduction of sympathetic autonomic action in almost all hemodynamic evaluations.

The comparative analysis between pupillometric and electromyographic variations is particularly interesting. The complexity of neurophysiological interactions in trigeminal proprioception, which are at the basis of the data registered in this case report, can permit us, at the moment, to hypothesize a different mode of activation of the LC-noradrenaline system. In habitual occlusion, the side with a greater myoelectric expressivity is commonly associated with the preferential side of mastication that determines a greater stimulation of periodontal receptors and increases of muscle splindle discharge frequency. These conditions can increase glutamate release, indirectly for the activation of presynaptic gamma-aminobutyric acid-A (GABA-_A_) receptors or directly through the trigeminal mesencephalic nucleus, or in the coerulean and peri-coerulean area [23]. The effects of changes in LC activity on autonomic functions result in complex patterns of neuronal interactions, because the LC exhibits pronounced responses also to non-noxious environmental stimuli [17,18], as in the case of muscle spindle and dental periodontal increases of discharge frequency. Moreover, preliminary findings showed that the LC and trigeminal systems released neuronal vasoactive peptides, as pituitary adenylate cyclase-activating peptide and vasoactive intestinal polypeptide [29,30]. LC and trigeminal asymmetric functions might have determined a concomitant asymmetrical release of these peptides into the autonomic system. In fact, the brainstem organization of the noradrenaline (NA) projections, coming from retrograde as well as anterograde transport studies, showed that the NA inputs to the trigeminal motor nuclei originate almost exclusively in A5 and A7 group cells with almost no contribution from the LC. By contrast, after the deposit of tracer in the rostral part of the spinal trigeminal nucleus, the majority of labeled NA neurons were found in LC [31]. The larger basal pupillometry and the better hemodynamics of the right side should confirm this hypothesis. The symmetrization of the periodontal proprioceptive input and the elimination of the occlusal open bite, both retrieved by the orthotic, may have induced a more appropriate and balanced reduction of the coerulean activity, together with a minor co-release of vasoactive peptides. The LC contribution to the control of autonomic activity results from direct projections to sympathetic divisions of the spinal cord, including the superior cervical ganglion which becomes innervated in the vertebral artery [15]. This datum could explain the larger and symmetric effects observed both on hemodynamic vertebral and basal pupillometric examinations.

In summary, if recent findings confirmed that the pattern and intensity of muscle contraction during voluntary working side chewing influenced the cerebral blood flow velocity, then it is also possible to affirm that the proprioceptive signals elicited by involuntary occlusion during swallowing can be important. Infact, the frequency of this act, even if for a short time (0.7 seconds), is constantly repeated every minute in 24 hours and can modify the hemodynamics of cerebro-afferent vessels as well as that of the sympathetic nervous activity. These effects could be interesting in that chronic cerebral hypoperfusion accelerates amyloid beta-deposition [32]. Hemodynamic parameters indicate a significant vertebral artery dysfunction and this could determine, in the long run, microcirculatory variations especially in reticular formation or/and in human cervical cord. These conditions may cause dizziness, drop attack, gait disturbance or cognitive impairment [33].

## Conclusions

Comparative data analysis has shown that an asymmetric involuntary occlusion may represent an interferential pattern on the functional modalities of LC, also involving the sympathetic autonomic systems with effects on pupillometric baseline and cerebral-afferent artery blood flow velocity. Within the limits of this case report, further investigations are necessary in order to detect the modalities, at the moment not completely proved, through which the occlusal proprioception may modulate the widespread LC-noradrenaline neuromodulator projections. As the pupillometric evaluations have proved useful to analyse the LC functional modes, it could be used as a diagnostic tool in the assessment of pathologies involving the LC-noradrenaline system, as cardiological and brain vascular stress-induced diseases. The inclusion of myoelectric masseter evaluations can be useful in patients with asymmetric hemodynamics of cerebro-afferent vessels and the trigeminal proprioceptive rebalance can integrate the complex therapy of patients with increased chronic sympathetic activity. Moreover, the repeated occlusal controls by computerized functional evaluations are essential both for orthodontic and dental prosthesis therapy and to prevent trigeminal proprioceptive unbalance-induced effects.

## Consent

Written informed consent was obtained from the patient for publication of this manuscript and accompanying images. A copy of the written consent is available for review by the Editor-in-Chief of this Journal.

## Competing interests

The author declares that he has no competing interests.
